# Indices to Measure Risk of HIV Acquisition in Rakai, Uganda

**DOI:** 10.1371/journal.pone.0092015

**Published:** 2014-04-04

**Authors:** Joseph Kagaayi, Ronald H. Gray, Christopher Whalen, Pingfu Fu, Duncan Neuhauser, Janet W. McGrath, Nelson K. Sewankambo, David Serwadda, Godfrey Kigozi, Fred Nalugoda, Steven J. Reynolds, Maria J. Wawer, Mendel E. Singer

**Affiliations:** 1 Rakai Health Sciences Program, Entebbe, Uganda; 2 Deparment of Epidemiology and Biostatistics, Case Western Reserve University, Cleveland, Ohio, United States of America; 3 Department of Epidemiology, Johns Hopkins Bloomberg School of Public Health, Baltimore, Maryland, United States of America; 4 Department of Anthropology, Case Western Reserve University, Cleveland, Ohio, United States of America; 5 Department of Epidemiology and Biostatistics, University of Georgia, Athens, Georgia, United States of America; 6 Makerere University College of Health Sciences, Kampala, Uganda; 7 Division of Intramural Research, National Institute of Allergy and Infectious Diseases, National Institutes of Health, Bethesda, Maryland, United States of America; 8 Johns Hopkins School of Medicine, Baltimore, Maryland, United States of America; University of New South Wales, Australia

## Abstract

**Introduction:**

Targeting most-at-risk individuals with HIV preventive interventions is cost-effective. We developed gender-specific indices to measure risk of HIV among sexually active individuals in Rakai, Uganda.

**Methods:**

We used multivariable Cox proportional hazards models to estimate time-to-HIV infection associated with candidate predictors. Reduced models were determined using backward selection procedures with Akaike's information criterion (AIC) as the stopping rule. Model discrimination was determined using Harrell's concordance index (c index). Model calibration was determined graphically. Nomograms were used to present the final prediction models.

**Results:**

We used samples of 7,497 women and 5,783 men. 342 new infections occurred among females (incidence 1.11/100 person years,) and 225 among the males (incidence 1.00/100 person years). The final model for men included age, education, circumcision status, number of sexual partners, genital ulcer disease symptoms, alcohol use before sex, partner in high risk employment, community type, being unaware of a partner's HIV status and community HIV prevalence. The Model's optimism-corrected c index was 69.1 percent (95% CI = 0.66, 0.73). The final women's model included age, marital status, education, number of sex partners, new sex partner, alcohol consumption by self or partner before sex, concurrent sexual partners, being employed in a high-risk occupation, having genital ulcer disease symptoms, community HIV prevalence, and perceiving oneself or partner to be exposed to HIV. The models optimism-corrected c index was 0.67 (95% CI = 0.64, 0.70). Both models were well calibrated.

**Conclusion:**

These indices were discriminative and well calibrated. This provides proof-of-concept that population-based HIV risk indices can be developed. Further research to validate these indices for other populations is needed.

## Introduction

Identifying individuals most-at-risk for HIV and targeting them with specific preventive interventions has been shown to be cost-effective [1]–[7]. These groups could be targeted with behavioral interventions combined with voluntary HIV testing and counseling (VCT), voluntary medical male circumcision (VMMC), and pre-exposure prophylaxis (PrEP) with antiretroviral drugs.

Moreover, the effectiveness of some preventive programs was shown to be higher among people most-at-risk of HIV. For example, male circumcision (MC) was more effective in studies of men deemed to be at high risk of HIV such as men recruited from clinics treating sexually transmitted infections and among truck drivers [8], or HIV uninfected men in discordant relationships with HIV-positive women [9]. Higher efficacy of MC was also suggested for men with many partners or genital ulcers in the Rakai circumcision trial [10].

With respect to PrEP, mathematical models suggest higher cost-effectiveness of oral PrEP among most-at-risk groups [11], [12].

Unlike countries where the HIV epidemic is limited to specific groups (concentrated epidemics) and high-risk groups are readily identified, in sub-Saharan Africa (SSA) with a generalized epidemic, identifying high-risk groups to target is especially challenging.

Prediction indices have been used successfully to predict the risk for chronic diseases such as coronary heart disease and cancer [13], [14] but have had limited application to HIV infection. To-date research into indices to predict HIV risk in SSA has been limited to discordant couple relationships [15], [16]. Development of indices for the general population is especially critical for SSA where identifiable discordant couple relationships contribute only modestly to HIV incidence [17], [18].

We developed gender-specific indices to predict the risk of HIV acquisition based on the general population of sexually active individuals who participated in the Rakai Community Cohort Study (RCCS) in Rakai district, Uganda.

## Methods

### Ethics statement

The Rakai Community cohort study (RCCS) was approved by the Science and Ethics Committee of Uganda Virus Research Institute, the Uganda National Council of Science and Technology and US-based Western IRB. Written consent was obtained from all research participants. Participants less than 18 years had their parents, caretakers, or guardians provide written consent for them in addition to their own written assent. Consent procedures were approved by the Science and Ethics Committee of Uganda Virus Research Institute, the Uganda National Council of Science and Technology and US-based Western IRB.

### Population

Derivation of the indices was based on data from the Rakai Community Cohort Study (RCCS), in Rakai district, South-Western Uganda. The cohort has been described previously [19], [20]. Briefly, the RCCS is an open population-based prospective cohort of approximately 15,000 consenting participants aged 15–49 years who were interviewed in surveys conducted every 12–20 months since 1994. Participants provided information in structured interviews and provided blood samples for HIV serology. The data collected included demographics, sexual behaviors, health and contextual characteristics. We used longitudinal data collected from 2003 to 2011. The maximum follow-up time was 7.7 years. The indices were limited to sexually active individuals who reported sexual intercourse in the previous 12 months.

### Potential predictor variables

The variables considered included participant demographics (age, marital status, education); sexual behaviors in the previous twelve months including number of sexual partners, frequency of condom use, use of alcohol before sex by either partner, casual sex, transactional sex, concurrent sexual partners and self-perception of exposure to HIV or perception of exposure by partner (for unmeasured risk factors); biomedical factors including genital ulcer symptoms, men's circumcision status, and use of hormonal contraception by women; HIV testing and counseling in the previous twelve months; contextual factors including community type (trading center versus village), whether one migrated to the community within the previous 2 years, Community HIV prevalence, whether or not the participant's employment type was associated with high risk of acquiring HIV); and partner characteristics including use of alcohol before sex, perception of partner's exposure to HIV, whether partner had a high risk employment type and whether the partner's HIV status was unknown. These factors have been associated with HIV risk in published literature [10], [21]–[31]. A full description of the variables and their coding is shown in table 1.

**Table 1:** Description of potential predictors, Rakai, 2003–2011.

Demographics	Type	Description
Age	Continuous	
Marital status	Categorical	Monogamous, polygamous, divorced/separated, never married
Education	Categorical	Primary/no education, at or above secondary level
**Behaviors**		
Number of sexual partners in last 12 months	Ordinal Categorical	1,2,3+ for males and 1,2+ for females
Frequency of condom use in last 12 months	Categorical	Always, sometimes and never
Alcohol before sex	categorical	Taking alcohol before sex, (Yes/No)
Casual sex	Categorical	sex with a non-regular, non-cohabiting partner (yes/no)
Transactional sex	categorical	Engagement in sex where money or gifts were exchanged as a condition for having sexual relations (Yes/No)
**Biomedical factors**		
Genital ulcers		Yes/No
Circumcision		Yes/No
Hormonal contraception		Taking hormonal pills, injectable, implanted hormonal contraceptives (Yes/No)
**VCT**		
HIV Testing in last 12 months	Categorical	Yes/No
**Sexual Partner variables**		
Used alcohol before sex	Categorical	As reported by partner (Yes/No)
Perceived partner's exposure to HIV	Categorical	As a response to the question: “How likely is it that your partner has been exposed to HIV, the virus that causes HIV/AIDS infection” Yes (very likely or likely), No (very unlikely or unlikely)
High risk employment	Categorical	Yes, if working in a bar, brewing alcohol, working in a restaurant, working in a hotel or guest house, fishing, truck or taxi driver, motor-cycle taxi rider (locally called boda-boda cyclist), market vending, housekeeper, trading which required one to work away from their community, and being in the army, police, or security work, No Otherwise
Unaware of partner's HIV status	Categorical	Yes/No
Domestic violence situation	Categorical	Involves violence received or perpetuated. (including verbal and physical insults)
**Contextual variables**		
Residence	Categorical	Village/Trading center
Recent immigration	Categorical	Yes/No (Moved to community within last 2 years)
Community HIV prevalence	Continuous	Prevalence of HIV in one's community

### Statistical methods

We performed frequency tabulations of potential categorical variables and summaries of continuous variables. We used a correlational matrix to determine pairwise correlations between potential variables. Highly correlated variables were combined if they measured similar domains of HIV risk. For this reason, use of alcohol before sex by oneself and one's sexual partner were combined into one variable. The same was done for self-perception of exposure to HIV and perception of exposure to HIV by sexual partners. Two percent of males and about three percent of females had at least one of the candidate variables missing. To avoid possible selection bias we imputed missing values using the aregImpute (Hmisc) R function. This function conducts multiple imputation by using the bootstrap samples for each of the multiple imputations and fits a flexible additive model on a sample with replacement from the original data and this model is used to predict all of the original missing and non-missing values for the variables being imputed [32].

We used Cox proportional hazards regression to model time-to-HIV infection as a function of candidate predictor variables. Since HIV infection was only detected at the time of the survey visits, the exact time of HIV infection was not known so the time of infection was assumed to be the middle of the interval between the last negative and first positive HIV test.

Analyses were stratified by gender because some of the important variables, such as circumcision status and use of hormonal contraception were gender-specific.

We assessed unadjusted associations between variables and HIV acquisition. To determine the optimal form for continuous variables (age and community HIV prevalence), we compared deviances of nested models of polynomials including linear, square, and cubic terms; as well as restricted cubic splines with 3–5 knots. As a result, age was modeled using a square term for men and a linear term for women. Community HIV prevalence was modeled using a linear term.

The global and variable-specific proportional hazards assumption was checked using the cox.zph R function [33] which examines the correlation coefficient between transformed survival time and scaled Schoenfeld residuals as well as the slope of the time-dependent coefficient.

In view of the evidence of higher effectiveness of male circumcision among high risk men [8], [10], we included an interaction term between number of sex partners and circumcision status in the full multivariable men's model. We used a main effects model for women. A reduced model was obtained using backward selection procedures with AIC as stopping rule. For statistical inference, the AIC criteria requires that the increase in model *χ* ^2^ for a given variable be greater than two times the degrees of freedom for the variable and a *χ* ^2^ p-value not greater than 0.157 for one degree of freedom [34], [35].

### Model performance

Model discrimination was assessed by using Harrell's concordance index (c index) for survival data and its 95 percent confidence interval [36]. We also examined discrimination graphically using a plot of cumulative HIV incidence by quartiles of predicted HIV risk score.

Model calibration was assessed graphically using a plot of 4-year observed versus predicted survival for groups of participants with different survival probabilities.

### Internal Validation

A bias-corrected (corrected for possible over-fitting) c index was obtained using bootstrap resampling validation procedures [36] with 200 bootstrap samples from the original sample. The bias-corrected c index gives the best estimate of discrimination if the coefficients from this model were applied to another sample to predict HIV acquisition.

### Proportion of new infections due to most-at-risk status

We determined the proportion of cumulative new infections contributed by the most-at-risk group. We used various thresholds of HIV risk scores to define most-at-risk status, including the upper quintile, upper two quintiles, upper quartile, upper third and upper half of the risk scores as possible thresholds.

The model was presented as a nomogram using Harrell's nomogram R function [37]. The nomogram provides scores associated with levels of predictors in the final model. Total points for a given subject are obtained by summing across scores from all variables.

Analyses used STATA 10.0 (StataCorp, College Station, TX) and R version 2.15.2 (The R Project for Statistical Computing).

## Results

### Sample

There were a total of 30771 participants in the RCCS surveys from 2003 to 2011. Of these, 4,181 were newly enrolled into the cohort at the last survey and so did not provide follow-up for outcome analyses. Of the remaining 26,590, other participants were excluded for the following reasons: 7,555 (28.4 percent) were seen only at one survey and so did not contribute to the incident outcome analyses; 2,620 (10.0 percent) were not sexually active during the study period; 2,013 (7.6 percent) were prevalent cases of HIV; and 1,122 (4.2 percent) had no HIV tests or had only one HIV test and therefore could not contribute to the outcome analysis. Therefore we used 13,280 initially HIV uninfected participants with follow up visits to develop the indices. Of these, 7,497 (56.4 percent) were women and 5,783 (43.6 percent) were men. The mean follow-up time was 4.0 years (SD = 1.98) with a maximum of 7.7 years.

A total of 567 incident cases of HIV occurred in this sample (incidence rate = 1.06 per 100 person years, 95% CI = 0.97–1.15); 342 among females (incidence = 1.11 per 100 person years, 95% CI = 1.00–1.24) and 225 among the males (incidence rate = 0.98 per 100 person years, 95% CI = 0.86–1.12)

Table 2 shows the distribution of baseline characteristics of the population, stratified by gender.

**Table 2:** Characteristics of Sexually active Participants, Rakai Cohort, 2003–2011.

	Men		Women	
	Number/Mean	Percent/SD	Number/Mean	Percent/Sd
All	5,783	100	7,497	100
Age (years, SD)	28.3	8.0	27.0	7.8
Marital status				
*Monogamous*	3,179	55.0	4,342	57.9
*Polygamous*	549	9.5	1,228	16.4
*Separated/Divorced*	242	4.2	778	10.4
*Never married*	1,813	31.3	1,149	15.4
Education				
*Primary/No education*	3,899	67.4	5,100	68.0
*Post-primary education*	1,884	32.6	2,397	32.0
Recent migrant to community	1,102	19.1	2,430	32.4
Community type				
*Village*	4,881	84.4	6,119	81.2
*Trading center*	902	15.6	1,378	18.4
Number of sexual partners				
*1*	3,308	57.2	7027	94.0
*2*	1,667	28.8	-	-
*2+*	-	-	446	6.0
*3+*	808	14.0	-	-
New sex partner	3,013	52.1	1,970	26.3
Transactional sex	145	2.5	43	0.5
Casual sex	662	11.5	111	1.5
Concurrent sexual partners	2,137	37.0	267	3.6
Frequency of condom use				
*Always*	862	14.9	593	7.9
*Sometimes*	2,215	38.3	1,692	22.6
*Never*	2,706	46.8	5,212	69.5
Alcohol use before sex	2,166	37.5	2,971	39.6
Genital ulcers	618	10.7	904	12.1
Perception of risk of exposure	1,691	29.2	3,011	40.2
Domestic violence	1,606	27.8	2,106	28.1
Circumcised	1,332	23.0	1332	23.1
Uncircumcised sexual partner	-	-	5212	69.7
Hormonal contraception	-	-	1522	20.3
VCT in last 12 months	1,852	32.0	2,935	39.2
Any partner of unknown HIV status	4,523	78.8	5,868	78.3
Partner in a high risk employment	636	11.0	3,083	41.1
Has high risk employment	1,986	34.3	973	13.0
Community HIV prevalence	11.7	3.6	11.9	3.8

### Time trends in HIV risk

We tested for time trends in HIV risk by comparing HIV incidence rates in two time periods 2003–2006 and 2007–2011, stratified by gender. 2003–2006 represented a period just before the roll-out of antiretroviral therapy and subsequent early roll-out (the peri-HAART period), and a period prior to the availability of male circumcision services. The period 2007–2011 was characterized by rapid scale-up of HAART and early roll-out of a male circumcision services in the Rakai communities. The difference in HIV incidence rate between the two time periods was not statistically significant. HIV incidence among men in the 2003–2006 period was 0.84per 100 person years (95% CI = 0.68–1.04) and 0.86 per 100 person years (95% CI = 0.71–1.03) in the second time period. The difference in incidence rate was −0.02 per 100 person years (95 percent CI = −0.20, 0.25). Among the females, HIV incidence in the 2003–2006 period was 0.99 per 100 person years (95% CI = 0.83–1.16) and 1.07 per 100 person years (95% CI = 0.92–1.23) in the second time period. The difference in incidence rate was −0.08 per 100 person years (95 percent CI =  is −0.30, 0.14)

### Unadjusted analyses

For men, all of the variables we tested were significantly associated with HIV acquisition in the unadjusted analyses at p≤0.157 (table 3) except high risk employment and recent migration to the community. For the women, non-significant variables included education, having a partner in high risk occupation, and hormonal contraception.

**Table 3:** Association between variables and HIV acquisition by Gender, Rakai, Uganda 2003–2011: Unadjusted analyses.

	Men			Women		
Characteristic	Relative Hazard	95% CI	p-value	RelativeHazard	95% CI	P-value
Age (linear, years)	1.27	1.05–1.44	0.002	0.98	0.96–0.99	0.001
Age (squared)	99.51	99.24–99.79	0.001	-	-	-
Marriage						
*Monogamous*	1	-	-	1	-	
*Polygamous*	1.38	0.90–2.12	0..140	1.01	0.79–1.52	0.569
*Separated*	2.42	1.47–3.98	0.001	2.73	2.05–3.63	<0.001
*Never married*	1.38	1.03–1.86	0.034	2.23	1.68–2.96	<0.001
Education						
*Primary/None*	1	-	-	1	-	
*Post-primary*	0.55	0.40–0.77	<0.001	0.88	0.69–1.11	0.284
Recent migration to community	1.12	0.79–1.59	0.522	1.50	1.20–1.88	<0.001
Community type						
*Village*	1			1		
*Trading center*	1.81	1.33–2.45	<0.001	1.24	0.96–1.62	0.102
Number of sexual partners						
*1*	1	-	-	1	-	-
*2*	1.36	1.00–1.86	0.053			
*2+*	-	-	-	3.96	2.99–5.25	<0.001
*3+*	2.71	1.97–3.72	<0.001	-	-	-
New sex partner	1.53	1.17–2.00	0.002	2.56	2.06–3.19	<0.001
Transactional sex	2.21	1.21–4.05	0.010	2.82	1.05–7.55	0.040
Casual sex	1.44	0.99–2.08	0.055	2.61	1.39–4.91	0.003
Frequency of condom use						
*Always*	1	-	-	1	-	-
*Sometimes*	1.20	0.81–1.78	0.363	1.25	0.83–1.88	0.276
*Never*	0.75	0.50–1.12	0.157	0.60	0.41–0.89	0.011
Concurrency	1.61	1.24–2.09	<0.001	4.26	3.07–5.93	<0.001
Alcohol use before sex	1.46	1.12–1.89	0.005	1.38	1.12–1.71	0.003
Perception of exposure to HIV	1.70	1.30–2.23	<0.001	1.75	1.42–2.16	<0.001
Genital ulcers	2.31	1.68–3.19	<0.001	1.96	1.49–2.56	<0.001
VCT in last 12 months	0.83	0.62–1.09	0.179	0.74	0.59–0.92	0.007
Circumcision	.56	0.39–0.82	0.003	-	-	-
Use of hormonal contraception	-	-	-	1.06	0.88–1.29	0.518
Partner of unknown HIV status	2.18	1.48–3.21	<0.001	1.44	1.09–1.90	0.011
High risk employment	1.17	0.89–1.52	0.262	1.65	1.26–2.16	<0.001
Partner in high risk employment	2.53	1.83–3.50	<0.001	1.09	0.88–1.35	0.425
Community HIV prevalence	1.05	1.01–1.08	0.003	1.04	1.01–1.07	0.002

### Multivariable analyses and final model discrimination

In the multivariable analysis 10 factors were selected in the men's model. These factors included age, education, circumcision status, number of sexual partners, alcohol consumption by self or partner, genital ulcers, being unaware of a partner's HIV status, community type, having a partner with a high-risk employment type and community HIV prevalence (table 4). Because age was modeled using a quadratic term, to obtain the effect of age, we differentiated the regression equation with respect to age and obtained the following equation: 
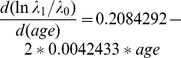
. This equation shows that the effect of age on the hazards of HIV acquisition is a function of age. The hazard increases more rapidly at age 15 and less rapidly thereafter and tends to reduce after 25 years of age. Men with post-primary education had 43 percent lower hazards of HIV infection compared to men with primary education or no education. Being circumcised was associated with 39 percent lower hazards compared to being non-circumcised. Compared to having one sexual partner in the previous 12 months, having two partners was associated with 21 percent higher hazards and 90 percent higher hazards for having three or more partners. Alcohol consumption before sex by self or partner was associated with 28 percent higher hazards. Having genital ulcers was associated with 81 percent higher hazards. Being unaware of a partner's HIV status was associated with 78 percent higher hazards. Living in in a trading center was associated with 67 percent higher hazards compared to living in the village. Having a partner with a high risk employment type was associated with 85 percent higher hazards. Every unit increase in community HIV prevalence was associated with three percent higher hazards.

**Table 4:** Multivariable Associations between variables and HIV acquisition by Gender, Rakai, Uganda 2003–2011.

	Men				Women			
	Hazard Ratio	95% CI	Coefficients	*χ* ^2^	HazardRatio	95% CI	Coefficients	*χ* ^2^
Age (linear)	1.23	1.04–1.44	0.2084	16.7	0.97	0.95–0.99	−0.0300	11.0
†Age (square)	99.57	99.30–99.86	−0.0042	8.8	-			
Marital status								
*Monogamous*	-	-	-	-	1	1	0	
*Polygamous*	-	-	-	-	1.10	0.79–1.54	0.0988	23.7
*Separated*	-	-	-	-	2.08	1.44–2.80	0.6987	
*Never married*	-	-	-	-	1.72	1.26–2.33	0.5404	
Education								
*Primary/Never*	1	-	0	11.7	1	-	0	
*Post-primary*	0.56	0.41–0.78	−0.5732		0.83	0.65–1.06	−0.1873	2.2
No of sexual partners								
*1*	1	-	0		1	-	0	
*2*	1.21	0.88–1.66	0.1879	14.2	-			
*2+*	-	-	-		1.59	0.97–2.63	0.4665	3.3
*3+*	1.90	1.36–2.67	0.6434		-			
New sex partner					1.45	1.09–1.92	0.3703	6.7
Either partner drunk alcohol before sex	1.28	0.96–1.71	0.2469	2.9	1.44	1.15–1.80	0.3617	9.9
Concurrent relationships					1.50	0.87–2.60	0.4058	2.2
Perception of HIV risk	-				1.49	1.20–1.86	0.4001	12.7
Gud	1.78	1.28–2.48	0.5939	12.5	1.75	1.33–2.31	0.5627	16.3
Circumcision	0.61	0.41–0.90	−0.4973	6.3	-	-	-	-
Community type								
*Village*	1	-	0	9.9	–	-	-	-
*Trading center*	1.67	1.21–2.31	0.5148		-	-	-	-
Partner with unknown HIV	1.82	1.22–2.70	0.5790	8.2	-	-	-	
Community HIV prevalence	1.03	0.99–1.07	0.0319	3.3	1.03	1.01–1.06	0.0369	7.7
Having a high risk occupation	-				1.32	0.99–1.75	0.2788	3.7
Partner in high risk occupation	1.89	1.34–2.67	0.6135	12.4	-	-	-	-

A similar reduced model for the women included the following 11 factors: age, marital status, education, number of sex partners, having a new sex partner, alcohol consumption by self or partner before sex, having concurrent relationships, being employed in a high-risk occupation, having genital ulcers, community HIV prevalence, and perceiving oneself or partner to have been exposed to HIV infection (table 4). Each year increase in age was associated with a three percent reduction in hazard of HIV acquisition. Post-primary education was associated with 17 percent lower hazards compared with primary education. Compared to monogamous marriage, being in a polygamous relationship was associated with 10 percent higher hazards of HIV acquisition, being separated or divorced was associated with two times the hazard, and women who were never married had 72 percent higher hazards. Compared to having one sexual partner in the previous 12 months, having two or more partners was associated with twice the hazards of infection. Having a new sexual partner in the previous 12 months was associated with 59 percent higher hazards. Concurrent relationships were associated with 50 percent higher hazards. Having genital ulcers was associated with 76 percent higher hazards. High risk employment type was associated with 32 percent higher hazards. Every unit increase in community HIV prevalence was associated with 3.8 percent higher hazards

### Discrimination

The final model for the men had a c index equal to 0.73 (95% CI = 0.69–0.75). After internal validation using bootstrap resampling the optimism-corrected c index was 69.1 percent (0.66, 0.73). The final model for the women had a c index equal to 0.69 (95% CI = 0.66–0.72) and the optimism-corrected c index after internal validation was equal to 0.67 (95% CI = 0.64, 0.70).

**Figure 1:**
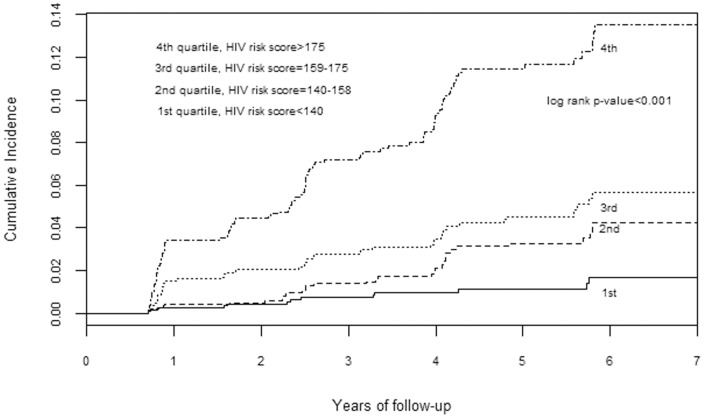
Cumulative HIV incidence by predicted quartile of HIV risk score for sexually active men.

**Figure 2:**
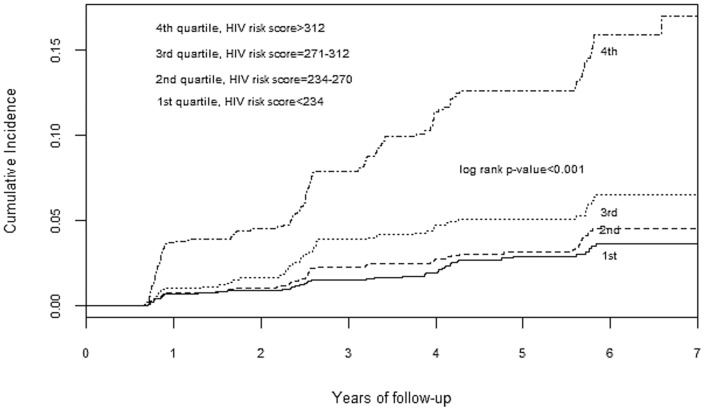
Cumulative HIV incidence by predicted quartile of HIV risk score for sexually active women.

Discrimination was also demonstrated graphically by plotting the cumulative HIV incidence by quartiles of risk score (a linear combination of products of model coefficients and levels of variables). The plot for men (figure 1) showed good separation of quartiles of risk especially the highest quartile. A similar trend is observed in the women's plot; however the separation is less for the lowest two quartiles (figure 2).

### Model calibration

**Figure 3:**
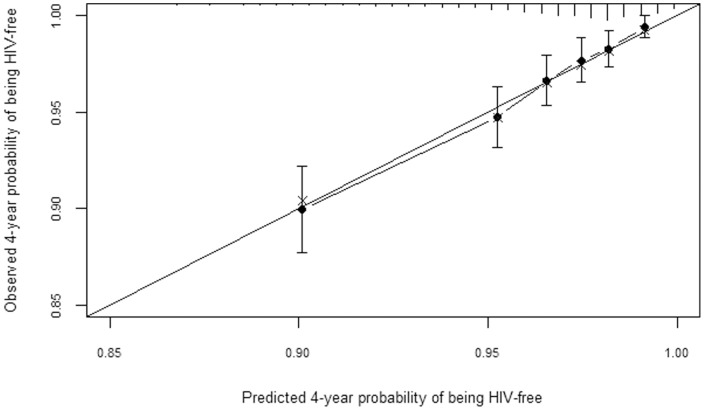
Observed vs predicted probabilities of being HIV-free at 4 years of follow-up. The figure provides bias-corrected calibration of the men's prediction model.

**Figure 4:**
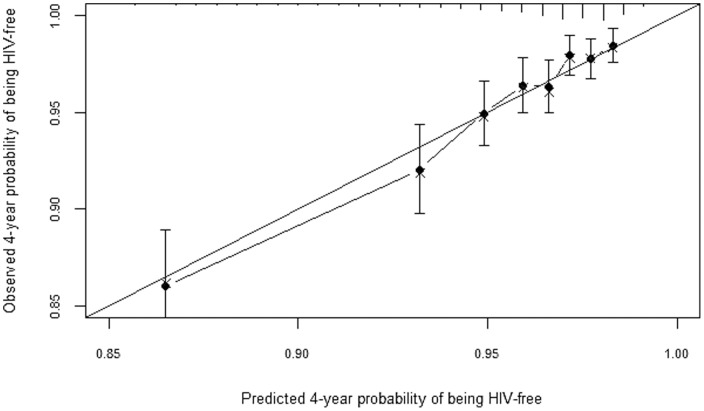
Observed vs predicted probabilities of being HIV-free at 4 years of follow-up. The figure provides bias-corrected calibration of the women's prediction model.

A plot of observed versus predicted probabilities of being HIV-free after four years of follow-up showed excellent agreement between observed and predicted probabilities (figures 3 and 4). Similar results were observed at two years of follow-up (results not shown).

### Nomograms

**Figure 5:**
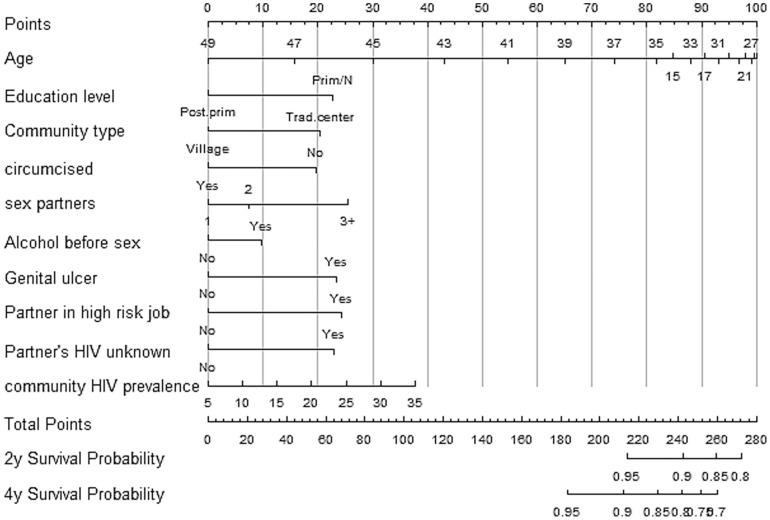
Nomogram of HIV risk for sexually active men developed using the Rakai cohort, Uganda 2003–2011.

**Figure 6:**
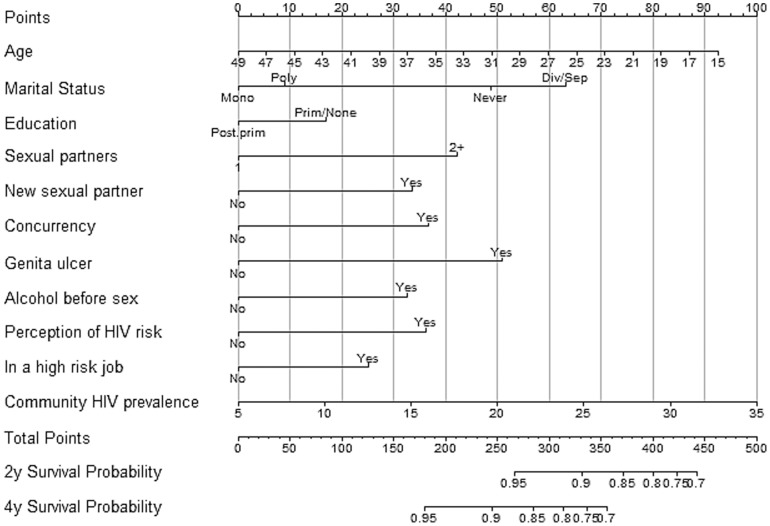
Nomogram of HIV risk for sexually active women developed using the Rakai cohort, Uganda 2003–2011.

Gender-specific nomograms are shown in figures 5 and 6. A score for each variable is obtained by drawing a vertical line to the top scale and reading off the variable's score for each individual. The total score for an individual is obtained by summing up all the individual variable scores. A scale for the total score and the corresponding 2-year and 4-year probabilities of being HIV-free are given at the bottom of the nomograms.

### Proportion of cumulative incidence due to most-at-risk status

In table 5 we provided proportions of cumulative incidence due to most-at-risk status at various thresholds. The upper quartile of nomogram scores contributed 55 percent of incident HIV among men and 48 percent among women; while the upper two quintiles contributed 70 percent among men and 63 percent among women. Proportions at other thresholds are shown in table 5.

**Table 5:** Proportion of Cumulative Incidence Contributed by Different Definitions of Most-at-risk status.

	Men			Women		
Threshold	Nomogram Score range	Proportion Cumulatve incidence(%)	95% confidence interval	Nomogram Score ange	Proportion Cumulatve incidence(%)	95% confidence interval
Upper quintile	>179	46.7	40.1–53.2	>325	40.1	35.3–45.8
Upper two quintiles	>164	70.2	64.2–76.1	>286	63.2	58.0–68.2
Upper quartile	>175	55.1	48.6–61.6	>314	48.0	42.7–53.2
Upper third	>169	63.1	56.7–69.4	>298	55.6	50.3–60.8
Upper half	>158	78.7	73.3–84.0	>271	68.7	63.8–73.6

## Discussion

Gender-specific indices to predict risk of HIV acquisition based on the Rakai cohort in Uganda were discriminative and well calibrated. Through graphical methods we showed that the indices showed better discrimination between the highest quartile of risk scores and lower quartiles. Also, most-at-risk groups defined by upper thresholds of risk scores contributed substantially to cumulative incidence (table 5). We believe that this property of the indices makes them suitable for identifying individuals most-at-risk of HIV infection.

To the best of our knowledge, this is the first effort to develop indices to predict individual risk of HIV infection in the general population of SSA. Our study has several strengths. We used a population-based longitudinal cohort which provided high quality data on known predictors of HIV acquisition. We tested various transformations of age to obtain the optimal transformation for age and community HIV prevalence. We also used self-perceived exposure or perceived exposure of partner to HIV as a predictor variable to capture unmeasured predictors of HIV infection. In this population self-perceived risk of exposure to HIV was associated with a higher risk of HIV infection [38].

Our study has limitations. All the variables apart from HIV prevalence were self-reported and therefore subject to recall error and social-desirability bias. However, these self-reported variables were found to be predictive of HIV risk. We believe that the ease of obtaining self-reports makes it feasible to use these indices in clinic settings or HIV counseling offices. We also did not validate these indices in other settings; however we noted that the indices performed well during internal validation; which provides a good indication that they are likely to perform well in other similar settings. However, despite the performance with internal validation, we recommend that these indices be externally validated before use in other populations similar to Rakai. To facilitate external validation, we have provided model coefficients in table 4. In settings where these indices may not be sufficiently predictive, techniques are available to re-calibrate them and update them for these news settings [39].

Given successful validation in other populations, these tools could be used in the context of voluntary counseling and testing to identify people most-at-risk of HIV for targeting them with preventive programs. An example of such interventions is oral pre-exposure prophylaxis (PrEP) with Tenofovir and Emtricitabine. In the FEM-PrEP [40] and VOICE (MTN 003) [41] trials, oral PrEP was not efficacious for HIV prevention due to low adherence to medications which was ascribed to low self-perceived risk of HIV [40]. Indices such as ours could help inform individuals of their true risk and thus offset false self-perceptions of risk. Therefore, these prediction indices, in addition to generating demand for HIV preventive services, could help maintain high levels of adherence to these services. Also, the routine use of these indices in HIV counseling may increase the efficiency of counseling by focusing on an individual's risk.

The implementation of these indices would require an HIV counselor to score clients HIV risk during a post-test counseling session using gender-specific nomograms provided in figures 5 and 6. The counselor would then determine the individual's level of HIV risk and provide appropriate risk-reduction counseling. We have provided a simple step-by-step guide in the online supplement to guide the implementation (supplement S1).

### Conclusion

We developed and internally validated gender-specific indices to predict risk of HIV infection. Our study provides proof-of-concept that indices to predict individual's risk of HIV infection can be developed to increase the efficiency of HIV prevention programs. Further research to validate these indices for other populations is needed.

## Supporting Information

Supplement S1 **A step-by-step guide to implementation of nomograms.**
(DOCX)
