# Supplementary material for: Indices to Measure Risk of HIV Acquisition in Rakai, Uganda
Source: PLoS One. 2014 Apr 4;9(4):e92015. doi: 10.1371/journal.pone.0092015 (PMC3976261; doi:10.1371/journal.pone.0092015)
Supplement: Supplement S1 — A step-by-step guide to implementation of nomograms. (DOCX) [file pone.0092015.s001.docx]

**Supplement to guide implementation of the nomograms**

These nomograms are strictly for use among persons who have been confirmed to be HIV-negative and have had sex in the past 12 months. The nomograms are gender-specific, so please make sure that you use the appropriate nomogram for the two different gender groups. The sections below provide procedures and instructions for implementation of the indices.

**Steps for implementation of the nomogram:**

Step 1: The nomogram has a reference line on top for reading scoring points. The points run from 0-100. Determine the client’s points for each predictor by drawing a straight line upwards to the reference line at the top. Determine the level for each individual client predictor by asking questions shown in the next section.

Step 2: Manually sum up the points for all predictors and assign the client a quartile of risk (from lowest to highest quartile) following the categorization for each gender group. The categorization for men is as follows:

Quartile 1: less than 140

Quartile 2: 141-158

Quartile 3: 159-175

Quartile 4: above 175

A similar categorization for females is as follows:

Quartile 1: less than 234

Quartile 2: 235-270

Quartile 3: 271-312

Quartile 4: above 312

Step 3: Proceed to provide individual counseling to the client about their risk for HIV.

*An example of instructions to a client:* “Mr. /Mrs. *X*, we are able to assess the risk of each client and place them in categories that correspond to their level of risk for HIV, The levels run from 1-4. Your risk for acquiring HIV in the future increases as you move from level 1 to 4. Our desire would be to have you at level 1. I will now proceed to ask you some questions to help me determine your level of risk and give you appropriate counseling thereafter.

**Question for men:**

1. *Have you had sexual intercourse with anyone in past 12 months?*

Instructions: If the response is “**NO”;** do **not** use the index. Continue to counsel client about the risk of HIV using the ABC strategy and other available prevention strategies. If the response is **“YES”,** proceed to ask questions to help with scoring the client.

1. Age: *How old are you?*
2. Education: *Have you ever gone to school? If yes, ask: To what level?* Categorize clients as post-primary or primary/no education.
3. Community type: *To you live in or within 3 km of a town/trading center or do you live in the village?*
4. Circumcision status: *Are you circumcised?*

**I would like to ask you some questions about you sexual life. The answers you give will be kept confidential and will be strictly used for this counseling session. I encourage you to be truthful.**

1. Number of sexual partners: *How many different sexual partners have you had in the last 12 months, including married or consensual partners?*
2. Alcohol with sex: *Did you or your partner drink alcohol before sex in the last 12 months?*
3. Genital ulcer: *Have you had a genital ulcer in the past 12 months or do you have one now?*
4. Partner in high risk employment: *Thinking about the partners you had in the last 12 months, what are/were theirs main occupations? (Please give me all their occupations*). **Instruction: check for high risk employment types** (including women employed in bars, restaurants, hotels or guest houses, fishing/fish mongers, market vending, housekeeping, trading which required one to work away from their community, and being in the army, police, or security work)
5. Unknown partner’s HIV status: *Thinking about all the partners you had sex with, is there any partner whose HIV status you do not know?*
6. **HIV prevalence of the community:** Please use the HIV prevalence of the community if it is known or use the district prevalence if the community prevalence is not known

**Questions for women**

1. *Have you had sexual intercourse with anyone in past 12 months?*

Instructions: If the response is “**NO”;** do **not** use the index. Continue to counsel client about the risk of HIV using the ABC strategy and other available prevention strategies. If the response is **“YES”,** proceed to ask questions to help with scoring the client.

1. Age: *How old are you?*
2. Marital status: *Are you currently married? If* ***No*** *ask: have you ever been married before?, If* ***yes****, ask whether her husband has other wives:* Categorize client as: never married, married monogamous, married in polygamous union, divorced/separated
3. Education: *Have you ever gone to school? If yes, ask: To what level?* Categorize clients as post-primary or primary/no education.

**Now I would like to ask you some questions about you sexual life. The answers you give will be kept confidential and will be strictly used for this counseling session. I encourage you to be truthful.**

1. Number of sex partners: *How many different sexual partners have you had in the last 12 months, including married or consensual partners?*
2. New sex partner: *Thinking about the partners you mentioned did you acquire any of them within the last 12 months?*
3. Concurrency: If she had more than one sex partner, ask: *You mentioned that you had X number of partners in the past 12 months; During the period you were having sexual relations with any one of them, did you also have sex with another partner/s or did these relationships come one after the other?* Score “yes” if relationships overlapped, otherwise score “No” if relationships were non-overlapping or if she didn’t have multiple relationships.
4. Genital ulcer: *Have you had a genital ulcer in the past 12 months or do you have one now?*
5. *Alcohol before sex: Did you or your partner drink alcohol before sex in the last 12 months?*
6. Perception of risk: How likely do you think that you have been exposed to HIV (the virus which causes AIDS)? Likely or unlikely
7. High risk employment type: What are your main occupations? **Instruction: check for high risk employment types** (including women employed in bars, restaurants, hotels or guest houses, fishing/fish mongers, market vending, housekeeping, trading which required one to work away from their community, and being in the army, police, or security work)
8. Thinking about all the partners you had sex with, is there any partner whose HIV status you do not know?
9. **HIV prevalence of the community:** Please use the HIV prevalence of the community if it is known or use the district prevalence if the community prevalence is not known

**Scores for each level of the Predictor**

**For men:**

Age Points

15 86

16 89

17 91

18 93

19 95

20 97

21 98

22 99

23 100

24 100

25 100

26 100

27 99

28 98

29 97

30 95

31 94

32 91

33 89

34 86

35 83

36 80

37 76

38 72

39 68

40 63

41 58

42 53

43 47

44 42

45 35

46 29

47 22

48 15

49 8

50 0

Education level Points

Prim/N 21

Post.prim 0

Community type Points

Village 0

Trad.center 19

Circumcised Points

No 18

Yes 0

sex partners Points

1 0

2 7

3+ 23

Alcohol before sex Points

No 0

Yes 9

Genital ulcer Points

No 0

Yes 22

Partner in high risk job Points

No 0

Yes 22

Partner's HIV unknown Points

No 0

Yes 21

community HIV prevalence Points

5 0

6 1

7 2

8 3

9 5

10 6

11 7

12 8

13 9

14 10

15 12

16 13

17 14

18 15

19 16

20 17

21 19

22 20

23 21

24 22

25 23

26 24

27 26

28 27

29 28

30 29

31 30

32 31

33 32

34 34

35 35

Total Points 2y Survival Probability

259 0.80

247 0.85

231 0.90

205 0.95

Total Points 4y Survival Probability

248 0.70

240 0.75

231 0.80

219 0.85

204 0.90

177 0.95

**For women**

Age Points

15 95

16 92

17 90

18 87

19 84

20 82

21 79

22 76

23 73

24 71

25 68

26 65

27 63

28 60

29 57

30 54

31 52

32 49

33 46

34 44

35 41

36 38

37 35

38 33

39 30

40 27

41 24

42 22

43 19

44 16

45 14

46 11

47 8

48 5

49 3

50 0

Marital Status Points

Mono 0

Poly 9

Div/Sep 63

Never 49

Education Points

Prim/None 17

Post.prim 0

Sexual partners Points

1 0

2+ 42

New sexual partner Points

No 0

Yes 33

Concurrency Points

No 0

Yes 37

Genita ulcer Points

No 0

Yes 51

Alcohol before sex Points

No 0

Yes 33

Perception of HIV risk Points

No 0

Yes 36

In a high risk job Points

No 0

Yes 25

Community HIV prevalence Points

5 0

6 3

7 7

8 10

9 13

10 17

11 20

12 23

13 27

14 30

15 33

16 37

17 40

18 43

19 47

20 50

21 53

22 57

23 60

24 63

25 67

26 70

27 73

28 77

29 80

30 83

31 87

32 90

33 93

34 97

35 100

Total Points 2y Survival Probability

445 0.70

425 0.75

402 0.80

374 0.85

335 0.90

270 0.95

Total Points 4y Survival Probability

358 0.70

339 0.75

316 0.80

287 0.85

248 0.90

183 0.95
